# A Text-Book of General Therapeutics

**Published:** 1894-03

**Authors:** 


*A Text-book of General Therapeutics.* By W. HALE WHITE, M.D. Pp. xi., 371. London: MACMILLAN & Co. 1889.

This excellent book has been but recently forwarded to us for review, although published more than four years ago. Without at this date venturing to enter upon detailed criticism, we can thoroughly recommend the work as far as it goes. Withal it is comprehensive enough, including in the subjects treated of such topics as climates, mineral waters, baths, non-medicinal antipyresis, electricity, hypnotism and some others, all well but not voluminously dealt with. Therapeutics exercised by means of drugs Dr. Hale White does not here include, as these are discussed in a separate work. Without going so far as to say that the book is the best in its own special line, yet it is so good as to make us wish that a new edition, thoroughly up to date, should very shortly make its appearance.

